# Specific Impact of Tobamovirus Infection on the Arabidopsis Small RNA Profile

**DOI:** 10.1371/journal.pone.0019549

**Published:** 2011-05-10

**Authors:** Quanan Hu, Jens Hollunder, Annette Niehl, Camilla Julie Kørner, Dalya Gereige, David Windels, Andreas Arnold, Martin Kuiper, Franck Vazquez, Mikhail Pooggin, Manfred Heinlein

**Affiliations:** 1 Botanical Institute, Department of Plant Physiology, Zürich-Basel Plant Science Center, University of Basel, Basel, Switzerland; 2 Department of Plant Systems Biology, Vlaams Interuniversitair Instituut voor Biotechnologie (VIB) - Ghent University, Ghent, Belgium; 3 Department of Plant Biotechnology and Genetics, Ghent University, Ghent, Belgium; 4 Institut de Biologie Moléculaire des Plantes du CNRS (UPR 2357), Université de Strasbourg, Strasbourg, France; 5 Department of Biology, Norwegian University of Science and Technology, Trondheim, Norway; Instituto Nacional de Câncer, Brazil

## Abstract

Tobamoviruses encode a silencing suppressor that binds small RNA (sRNA) duplexes *in vitro* and supposedly *in vivo* to counteract antiviral silencing. Here, we used sRNA deep-sequencing combined with transcriptome profiling to determine the global impact of tobamovirus infection on *Arabidopsis* sRNAs and their mRNA targets. We found that infection of *Arabidopsis* plants with *Oilseed rape mosaic tobamovirus* causes a global size-specific enrichment of miRNAs, ta-siRNAs, and other phased siRNAs. The observed patterns of sRNA enrichment suggest that in addition to a role of the viral silencing suppressor, the stabilization of sRNAs might also occur through association with unknown host effector complexes induced upon infection. Indeed, sRNA enrichment concerns primarily 21-nucleotide RNAs with a 5′-terminal guanine. Interestingly, ORMV infection also leads to accumulation of novel miRNA-like sRNAs from miRNA precursors. Thus, in addition to canonical miRNAs and miRNA*s, miRNA precursors can encode additional sRNAs that may be functional under specific conditions like pathogen infection. Virus-induced sRNA enrichment does not correlate with defects in miRNA-dependent ta-siRNA biogenesis nor with global changes in the levels of mRNA and ta-siRNA targets suggesting that the enriched sRNAs may not be able to significantly contribute to the normal activity of pre-loaded RISC complexes. We conclude that tobamovirus infection induces the stabilization of a specific sRNA pool by yet unknown effector complexes. These complexes may sequester viral and host sRNAs to engage them in yet unknown mechanisms involved in plant:virus interactions.

## Introduction

RNA silencing is a sequence-specific mechanism that coordinates the expression, protection, stability, and inheritance of eukaryotic genomes. It is involved in tuning critical developmental, stress-responses, and bodyguard functions by regulating the expression of genes at the transcriptional and post-transcriptional levels, or by triggering the formation of heterochromatic DNA regions [1], [2], [3]. RNA silencing is mediated by 21–24 nt small RNAs (sRNAs) that are processed from long dsRNA by RNase III enzymes of the DICER family (DICER-Like - DCL in plants). These sRNAs are classified into small interfering RNAs (siRNAs) and microRNAs (miRNAs) depending on their origin [2], [4], [5]. siRNAs and miRNAs associate with proteins of the ARGONAUTE (AGO) family to form RNA-Induced Silencing Complexes (RISC) in which they serve as guides to complementary RNA or DNA targets [4], [6]. AGO-containing RISCs can then mediate degradation of complementary endogenous or viral RNAs, translational repression of mRNAs, or transcriptional silencing of transposons and DNA repeats.

Plants encode several members of these protein families. For instance, the *Arabidopsis thaliana* genome contains four DCL and ten AGO genes. Several sRNA classes that depend on different pairs of DCL and AGO proteins have been identified. For example, whereas DCL1-dependent miRNAs guide AGO1, AGO7, or AGO10 to corresponding target RNA transcripts, DCL3-dependent siRNAs guide AGO4, AGO6 or AGO9 to DNA targets [7], [8], [9], [10], [11], [12], [13], [14]. During their transfer from DCL proteins to AGOs, sRNAs are stabilized by methylation of their 3′ terminal nucleotide by HEN1, which provides protection against oligo-uridinylation and degradation by nucleases of the SDN family [15], [16], [17]. Loading of sRNAs into AGOs appears to be restricted by DCL-AGO interactions and to depend at least in part on the identity of the first 5′ nucleotide of the sRNAs [14], [18], [19]. Thus, 21-nt sRNAs with 5′-terminal uridine (5′U) are predominantly bound to AGO1 and, in one specific case, to AGO7, with 5′A to AGO2, and with 5′C to AGO5, whereas 24-nt sRNAs with a 5′A are bound to AGO4. It is still unknown which of the remaining AGOs (if any) could preferentially bind sRNAs with 5′G.

RNA silencing is known to play a critical role in defense against viruses in plants and insects [20], [21], [22]. Thus, viral RNAs are used by the RNA silencing machinery to generate viral sRNAs (vsRNAs) that can potentially be loaded into specific AGOs to further target viral RNAs for cleavage and degradation or for translational repression. As part of the ongoing host-virus arms race, viruses have evolved potent RNA silencing suppressors (VSRs). As they evolved independently, the VSRs of different viruses inhibit different RNA silencing pathway components [23], [24]. We and others have shown that *Tobacco mosaic virus* (TMV) and related tobamoviruses encode a VSR that resides in the small subunit of their replicase [25], [26], [27]. This subunit binds siRNA and miRNA duplexes *in vitro* and interferes with their methylation [25], [26], [28], [29], a modus operandi that was also reported for several other VSRs like the Hc-Pro of *Tobacco etch virus* (TEV) or p19 of tombusviruses [30]. Consistently, miRNAs levels are generally increased in plants infected with TMV, TMV-Cg, cr-TMV, or *Oilseed rape mosaic virus* (ORMV)[25], [26], [29], [31], [32]. Studies of few cases have demonstrated that the increased miRNA levels triggered by tobamovirus infection results in increased levels of cognate mRNA targets [25], [31] and suggested that sRNA binding by tobamovirus replicase interferes with RISC loading or activity. This model is supported by the ability of cr-TMV suppressor protein to bind sRNA duplexes and to inhibit RISC assembly *in vitro* [25]. However, a recent report indicates that a positive correlation between miRNA enrichment and increased mRNA target levels may not be necessarily mediated by miRNA sequestration. Thus, it was shown that the enrichment of miR168 in plants infected with *Cymbidium ringspot virus* occurs through an activity independent of binding by the VSR p19 and that miR168 functions in translational repression rather than in mRNA cleavage despite of the increased AGO1 mRNA levels [33]. Moreover, the movement protein (MP) of TMV enhances the spread of silencing [34] suggesting that the impact of tobamoviruses on the sRNA profile and gene expression may not be limited to infected cells but may be able to spread ahead of the leading front of infection. These examples clearly indicate that further studies are needed to understand the impact of virus infection on the host sRNA profile and gene expression and its role in virus susceptibility and/or defense.

So far our knowledge on changes of sRNA and transcriptome profiles induced by a virus in its host is limited. Our current knowledge is based on the analysis of a limited number of sRNAs and of their targets by Northern blots or on small-scale sRNA cloning [25], [31]. Analysis of the full sRNA complement of cells can be achieved by deep sequencing using a variety of available platforms [35] and first reports describing changes in the plant sRNA profile in response to pathogens are appearing [36]. While deep sequencing analyses of virus-infected plants allowed to describe the profile of virus-derived siRNAs [37], [38], [39], [40], [41], [42], a comprehensive view on the global impact of virus infection on the profile of host plant sRNAs and their mRNA targets is still lacking.

We have used a combination of mRNA profiling and sRNA deep sequencing to understand the impact of ORMV infection on the Arabidopsis sRNA and transcriptome profiles. We describe here a global analysis of viral sRNAs and the changes in cellular sRNA profiles during systemic infection. Our analysis shows that virus infection changes the pattern of sRNAs that are processed from miRNA and ta-siRNA precursors. The virus-induced sRNA profile is inconsistent with the proposed stabilization of sRNA duplexes by the replicase as a sole mechanism and suggests that the observed changes in the sRNA profile involve additional mechanisms. In particular, we found that ORMV infection leads to the specific enrichment of 21 nt sRNAs with a 5′ terminal guanine. This suggests that these sRNAs associate with size-specific and 5′ nucleotide-specific effector complexes of yet-unknown nature. Our mRNA profiling data demonstrate that increased levels of miRNAs and siRNAs do not correlate with significant changes in target transcript levels indicating that the virus-induced sRNA fraction is sequestered or active at different levels. Virus infection also leads to the accumulation of novel miRNA-like siRNAs (ml-siRNAs) encoded by miRNA precursors that might be part of a specific plant response to pathogens.

## Results

To gain insights into the effects of ORMV infection on the Arabidopsis sRNA profile we conducted Illumina sequencing of sRNA populations extracted from ORMV-infected and mock control-inoculated Arabidopsis Col-0 plants at 7 days post-inoculation (dpi). Following removal of adapter sequences the reads were mapped to the virus and *Arabidopsis thaliana* genomes (Figure S1, A) and only the reads with perfect match were further analyzed. Of 1,787,490 mapped sRNA reads found in the virus-treated sample, 80.1% (1,431,362 reads) originated from *Arabidopsis* and the remaining 19.9% (356,128) of the reads originated from the virus (Figure S1, A and B). Consistent with their biogenesis by DCL4 [29] ORMV vsRNAs are predominantly 21 nt in length (Figure S1, C). The vsRNAs map all along the viral genome, with 88.1% being homologous to the positive strand and 11.9% to the negative strand (Figure S1, E). A similarly strong positive strand bias in vsRNA accumulation has also been reported in the case of TMV-Cg infected samples [40] and may reflect the strong (+)strand-specific accumulation of viral RNA in infected cells [43], [44]. The origin of the conspicuous vsRNA hotspots along the viral genome is yet unknown but may be caused by predominant DCL cleavage of structured, double-stranded RNA regions.

### ORMV infection causes size-specific and 5′nucleotide-specific enrichment of plant sRNAs

**Figure 1:**
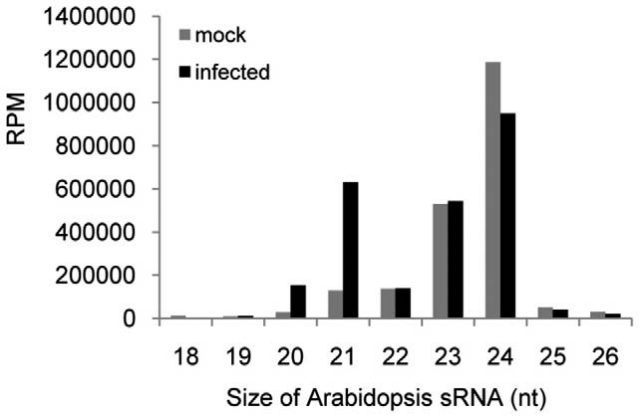
Size-distribution of Arabidopsis sRNAs in ORMV-infected and non-infected plants.

Comparison of the profiles of the mapped Arabidopsis-encoded sRNA obtained for mock and virus-treated samples reveals a significant impact of virus infection. The normalized, size-specific distribution of sRNAs shows a significant decrease in the proportion of 24 nt sRNAs, whereas the proportion of 21 nt sRNAs is increased (Figure 1). Consistently, there is a reduction in the proportion of sRNA reads derived from transposons and centromeric regions, whereas the proportion of reads for miRNAs and ta-siRNAs is increased 4.5-fold (Table 1).

**Table 1:** sRNA reads* mapped to the *Arabidopsis* genome.

	Mock		Infected	
Type	Unique Reads	Total Reads	Unique Reads	Total Reads
Known miRNA precursors	380	48514	1032	276483
Known TAS precursors	1220	10195	2425	48303
Gene	42003	133451	59901	236165
Tandem repeats	22559	76359	26261	81339
Inverted repeats	14560	66799	19287	78722
Transposons	70568	126655	67768	113376
Centromeric region	2160	11248	2627	10113
rRNA, tRNA, snoRNA and snRNA	8486	98765	10386	161872

*Reads are RPM

**Figure 2:**
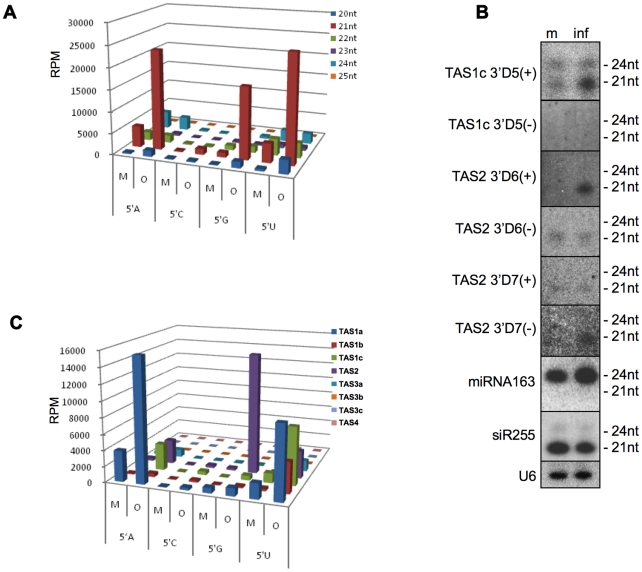
Effect of ORMV infection on the ta-siRNA profile. (**A**) ta-siRNA reads for ORMV-infected (O) and non-infected (mock-treated, M) plants are sorted according to size (nt) and 5′ terminal nucleotide. Although ta-siRNAs occur in different sizes, only 20 nt and 21 nt ta-siRNAs are enriched in ORMV-infected plants. Enriched ta-siRNAs have a 5′A, 5′U, or 5′G. (**B**) Northern blots confirming the size-specific enrichment of ta-siRNAs in infected (inf) compared to mock-treated (m) plants. The enrichment of ta-siRNAs in infected tissues (inf) is also strand-specific – usually, only one strand of the DCL-processed ta-siRNA duplex is enriched. The enrichment of miR163 suggests that size selection occurs at the duplex level just after cleavage by DCL (details in the text). The TAS1a/b/c-derived siR255 is not enriched, as has been previously reported. (**C**) 21 nt long ta-siRNA reads for ORMV-infected (O) and non-infected (M) plants sorted according to their 5′ terminal nucleotide and the TAS genes from which they are derived. ta-siRNAs with a 5′ terminal A or U are derived from different TAS genes, whereas ta-siRNAs with a 5′G are exclusively derived from the TAS2 gene.

Arabidopsis ta-siRNAs are derived from four families of *TAS* genes. The biogenesis of ta-siRNAs is initiated by AGO-mediated cleavage of *TAS* transcripts guided by miR173 (*TAS1a/b/c* and *TAS2*), miR390 (*TAS3a,b,c*), or miR828 (*TAS4*) [14], [45], [46], [47], [48]. Cleaved transcripts are then converted by RDR6 to long dsRNAs that are subsequently processed by DCL4 into phased ta-siRNAs that are in register with the cleavage site [45], [47], [49], [50], [51]. The majority of ta-siRNAs sequenced in both of our datasets were derived from *TAS1a,b,c* and *TAS2* loci (Table S1). Interestingly, although *TAS* family loci in Arabidopsis produce ta-siRNAs of different sizes, as observed previously [52], only the 20 and 21 nt ta-siRNAs are significantly enriched (6.5. and 7.5 fold, respectively) in ORMV-infected plants (Figure 2A, Table S1). To confirm this size-specific enrichment, we analyzed the sRNAs from mock- and virus-treated samples by RNA blot hybridization using several probes detecting ta-siRNAs of different sizes and from different phases [47], [53]. Figure 2B shows for TAS1c 3′D5(+) that infection leads to the enrichment of the 21-nt siRNA species, whereas the level of the 24 nt siRNA species remains constant. The blots confirm that the enriched ta-siRNA species can be derived from either strand of the *TAS* RNA duplex. For example, whereas TAS2 3′D6(+) siRNA strand is enriched in infected tissues, the complementary strand (TAS2 3′D6(-)) of the ta-siRNA duplex is not. Conversely, specific enrichment is observed for the TAS2 D7 (-) siRNA strand, whereas the level of the complementary TAS2 3′D7(+) strand remains unchanged. Consistent with previous observations [25], [29], the level of siR255, which derives from *TAS1a,b,c* and other loci, does not show any increase upon virus infection.

Since the loading of AGO proteins depends on the identity of the first 5′ nucleotide of sRNAs, we analyzed the distribution of the 5′ nucleotide of the enriched sRNA pool. Figure 2A shows that the degree of over-representation of 21 nt ta-siRNAs caused by ORMV-infection differs according to the 5′ terminal nucleotide in the order G>A = U>C (Figure 2A). Interestingly, we found a correlation between the 5′ nucleotide-specificity of the 21 nt ta-siRNAs and the specific precursor *TAS* RNAs from which they are derived. Specifically, enriched ta-siRNAs with a 5′G are predominantly derived from *TAS2* including the validated 3′D6(+) and 3′D7(-) species (Figure 2B and C), although as for the other *TAS* loci, the unique TAS2 ta-siRNAs with a 5′G (and also those with a 5′C) are underrepresented compared to ta-siRNAs starting with A or U (Table S2). It should be noted, however, that size- and 5′-terminal guanine are unlikely to represent sufficient criteria to dictate sRNA enrichment since some 21-nt siRNAs initiating with a 5′G are not enriched upon ORMV infection (data not shown). Size-specific selection may occur at the level of the sRNA duplex, since infection enriches 24 nt long miRNA163. Although this sRNA is 24 nt in length, it assumes a shorter physical length upon forming a bulge when paired with its 21 nt long passenger strand. Thus, the presence of miRNA163 as a duplex may represent an important prerequisite for its selection and subsequent stabilization in ORMV-infected plants.

**Figure 3:**
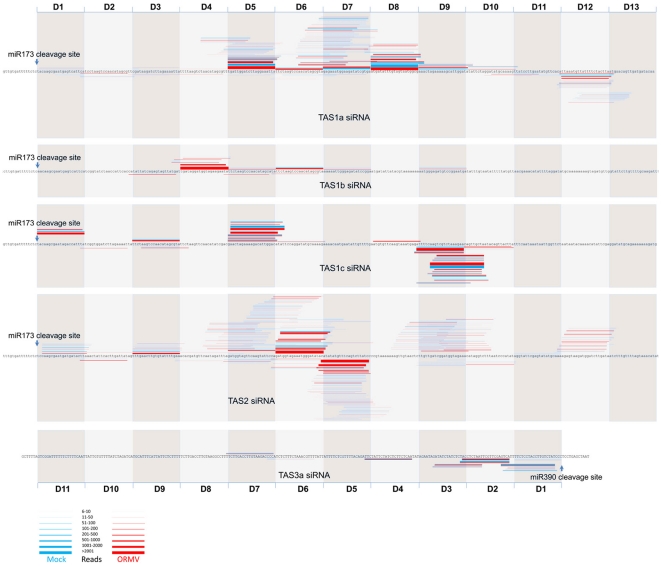
ta-siRNAs mapped to *TAS* transcripts. The *TAS* transcripts, the sites of miRNA cleavage, and the phased ta-siRNA windows are shown. Mapped unique ta-siRNA reads present in non-infected plants (Mock) and ORMV infected plants (ORMV) are shown as blue and red bars, respectively. Bar thickness indicates the number of reads for each unique ta-siRNA. ORMV infection does not change the phasing and complexity of the unique ta-siRNA population; only the frequency of the unique ta-siRNAs is changed. ORMV infection does not affect initial miRNA-guided *TAS* mRNA cleavage.

To test whether infection has an effect on the phased processing of ta-siRNAs, we mapped the frequency of unique ta-siRNA species with more than 5 reads to *TAS1a*, *TAS1b*, *TAS1c*, *TAS2*, and *TAS3a* genes (Figure 3). A change in ta-siRNA phasing was not observed indicating that the initial miRNA-guided cleavage mediated by AGO1 (*TAS1/2*) and AGO7 (*TAS3a*) and subsequent processing of the *TAS* RNA duplexes by DCL4 are not affected in infected cells. The distribution of the mapped ta-siRNA reads mapped to the *TAS* genes confirms that the enrichment is restricted to specific ta-siRNAs and that in most cases only one of the two strands of the processed ta-siRNA duplexes is enriched. Collectively, these observations indicate that the virus-induced enrichment of ta-siRNAs occurs at a post-processing step, presumably through stabilizing associations with specific effector complexes, as is suggested by the strand, size and nucleotide specificity of the ta-siRNAs.

We note that the specific enrichment of 20–21 nt siRNAs is not restricted to *TAS* loci. A similar effect is indeed also observed for other RDR6/DCL4-dependent, secondary siRNA-generating loci [52] (Table S3) or for IR71, which is one of the long inverted repeats of Arabidopsis that generates all size classes of siRNAs in an RDR-independent fashion [53], [54], [55], [56], [57] (Table S4).

**Figure 4:**
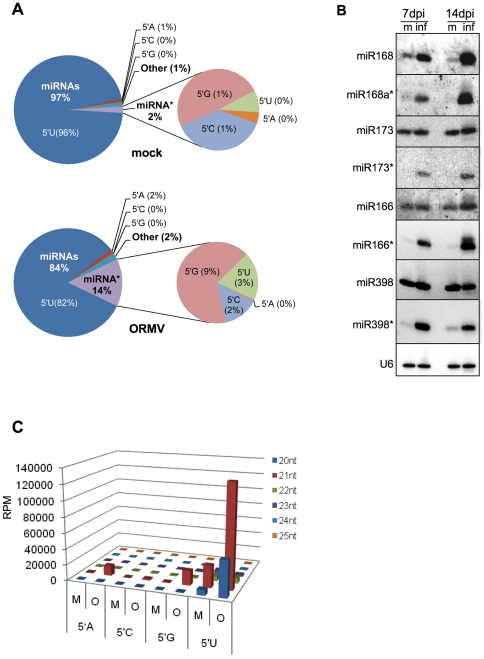
Effect of ORMV infection on the miRNA profile. (**A**) ORMV infection produces a significantly higher fold-change in the levels of miRNA* sequences (miRNA passenger strands) than in the level of miRNAs. The virus-induced fold-change is strongest for miRNA* sequences carrying a 5′ G nucleotide. (**B**) Northern blots confirming the virus-induced enrichment of miRNAs and the much stronger enrichment for their corresponding miRNA* sequences. m, mock; inf, infected; dpi, days post inoculation. (**C**) Normalized miRNA reads (RPM) for ORMV-infected (O) and non-infected (mock-treated, M) plants sorted according to size (nt) and 5′ terminal nucleotide. The majority of miRNAs starts with a 5′ U nucleotide and these miRNAs are strongly enriched in infected plants. The virus-induced peak of sRNAs starting with a 5′G is mostly due to enriched miRNA* sequences (as seen in **A**).

Next, we investigated the impact of virus infection on the profile of miRNAs. Previous studies using specific Northern blot probing or small-scale sequencing indicated an enrichment of miRNAs in tobamovirus infected tissues [25], [26], [31]. The results of our global profiling analysis confirm this trend. We found that all but four of the 35 miRNA families with sequencing reads in both samples showed an increased number of reads upon infection (Table S5). Interestingly, virus infection caused a much higher increased accumulation of miRNA* sequences compared to that of the corresponding miRNAs. Thus, whereas the sRNAs derived from miRNA precursor RNAs in mock-treated plants comprise 97% miRNAs and only 2% miRNA passenger strands, the relative amount of miRNAs in ORMV-infected plants is reduced to 84% whereas that of miRNA* sequences is increased to 14% (Figure 4A). Generally, the number of reads and the degree of accumulation in virus-infected plants differs between miRNAs and their passenger strands (Fig. S5). These observations were confirmed by sRNA blot hybridizations (Figure 4B). Further analysis showed that this strong enrichment of miRNA* sequences primarily concerns those initiating with a 5′G resulting in miRNA* sequences with a 5′G as the predominant miRNA* species in infected cells (Figure 4A). A summary of the virus-induced enrichment of miRNA gene-derived sRNAs is shown in Figure 4C. The increase in accumulation seen for sRNAs initiating with a 5′U is to the majority caused by virus-enriched miRNAs, whereas the increase seen for sRNAs starting with a 5′G reflects the accumulation of miRNA* sequences (see also Figure 4A). The passenger strands of miRNA160, miRNA396, and miRNA398 exhibit the strongest contribution to the increased pool of miRNA* sequences starting with a 5′G in virus-infected plants (highlighted in Table S5). However, a 5′G is not the only determinant for enrichment of passenger strands. For example, strong increases in accumulation are also shown by the passenger strands of miRNA408 and miRNA472, although these miRNA* sequences initiate with a 5′C and 5′U, respectively (Table S5). Nevertheless, the overaccumulation of miRNA* strands compared to miRNA guide strands and the preference for 5′G-miRNA* is again indicative of specific sRNA-associated effector complexes formed upon virus infection.

### Effect of ORMV infection on the host plant transcriptome

**Figure 5:**
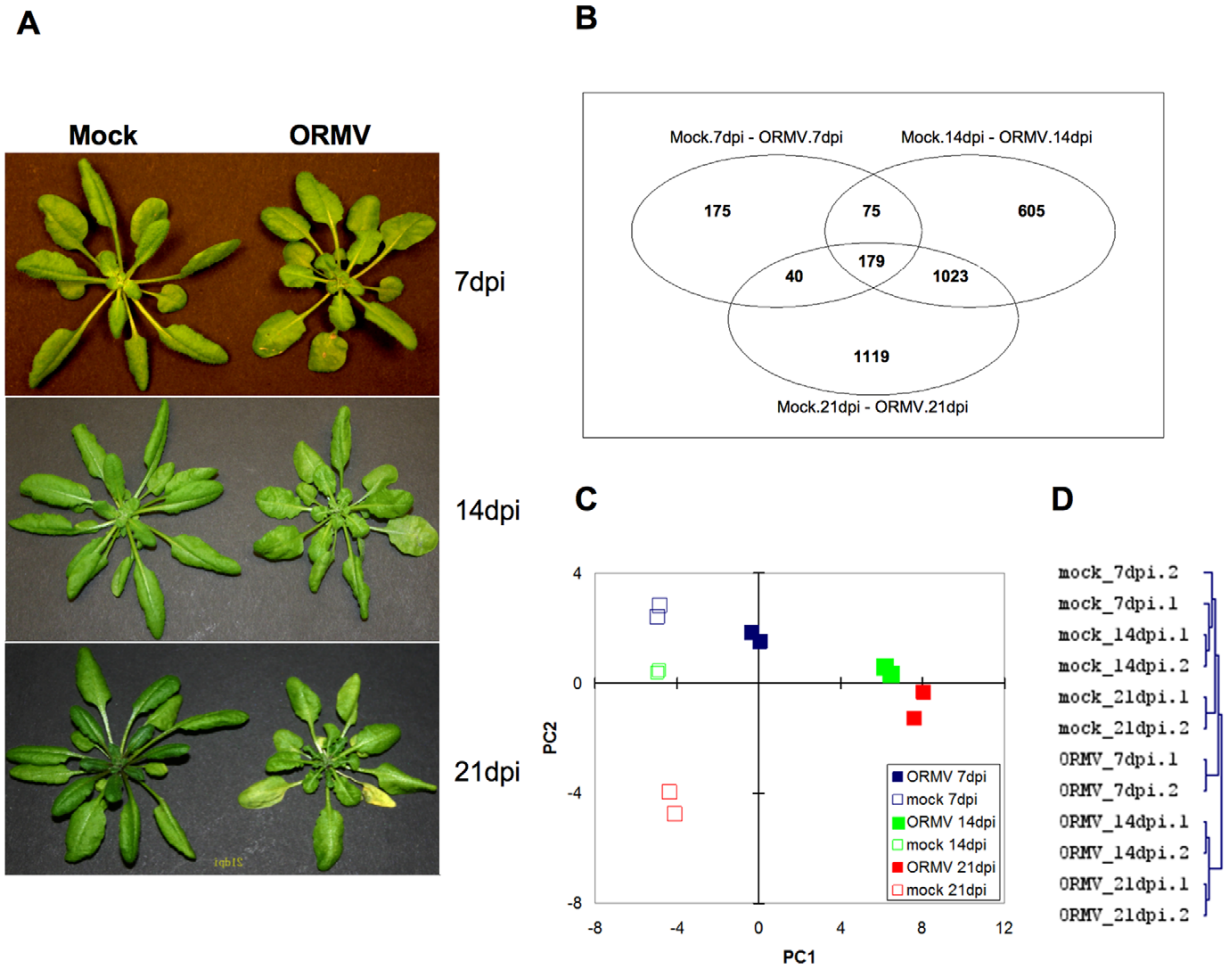
Effect of ORMV infection on the Arabidopsis transcriptome. (**A**) Disease symptoms of ORMV-infected plants as compared to mock-inoculated, non-infected plants at 7, 14, and 21 dpi. (**B**) Venn-diagram depicting the genes differentially expressed upon ORMV infection. A log_2_ fold-change cut-off value of 2 and a significance threshold of 0.001 were used for data analysis. (**C**) Principal component analysis on RMA-normalised expression values illustrating reproducibility between specific profiles and a clear data separation between the specific treatments. (**D**) Hierarchical clustering of expression values.

Although ORMV starts to spread systemically within 2–3 days after inoculation of individual leaves (Niehl and Heinlein, unpublished), viral symptoms are only observed after 10 days. From this time onward the newly emerging leaves and progressively also the older leaves show curling and retarded growth, and the oldest leaves show signs of necrosis. Three weeks after inoculation, the plants show a clear growth retardation phenotype (Figure 5A).

To test whether the significant virus-induced changes in the sRNA profile seen at 7 dpi correlate with significant effects on transcript levels, we profiled mRNA transcripts at 7, 14, and 21 dpi using Affimetrix ATH1 arrays. In this time-course experiment we observed a gradual increase in accumulation of ORMV genomic RNA and vsRNAs (data not shown) and a gradual increase in symptom severity (Figure 5A). The total RNA extracts for profiling transcripts at 7 dpi were the same as those used for sRNA deep sequencing described above and the extracts for the later time points were prepared and processed exactly the same way. The Venn diagram of RMA-normalized data (Figure 5B) highlights the 3216 genes differentially expressed upon ORMV infection with a log_2_ fold-change cut-off of 2 and a significance value of p<0.001. Of those, 175, 605, and 1119 genes displayed differential expression in virus-infected compared to mock-inoculated samples exclusively at 7, 14 and 21 dpi, respectively. 179 genes were differentially expressed upon ORMV infection at all the three time points. In addition, 75 genes were regulated upon ORMV infection at the two earlier time points (7 dpi and 14 dpi), 40 genes were differentially expressed upon ORMV infection only at 7 dpi and 21 dpi, and 1023 genes exhibited differential expression at the two later time points (14 dpi and 21 dpi). Independent samples were used for qPCR analysis, which reproduced the infection-induced changes in the expression of the tested genes (data not shown). The quality of the data was also validated by principal component analysis (PCA, Figure 5C) and hierarchical clustering (Figure 5D), indicating that the replicates show very similar responses and that the datasets for mock-inoculated and virus-infected samples are clearly separated. Importantly, for the virus-infected samples, the 7 dpi time point is clearly distinct from the later time points. This suggests that expression changes at 14 and 21 dpi may be related to secondary effects that are related to tissue crinkling and chlorosis, whereas the earlier time point reflects more specific responses to the virus infection and its spread.

Functional GO term enrichment analysis (log_2_>2; p<0.001) reveals that at 7 dpi there are responses of gene classes related to responses to biotic stimuli, other organisms, stress, defence, and immune system processes, whereas responses at the later time points are focussed to gene classes rather responding to metabolic processes and abiotic stimuli (Table S6). This finding may indicate that at late time points, when the virus has accumulated to high levels, secondary effects on the plant metabolism and nutritional status play a role in addition to the responses to virus.

### Effect of ORMV infection on mRNA targets of miRNA and ta-siRNA

**Figure 6:**
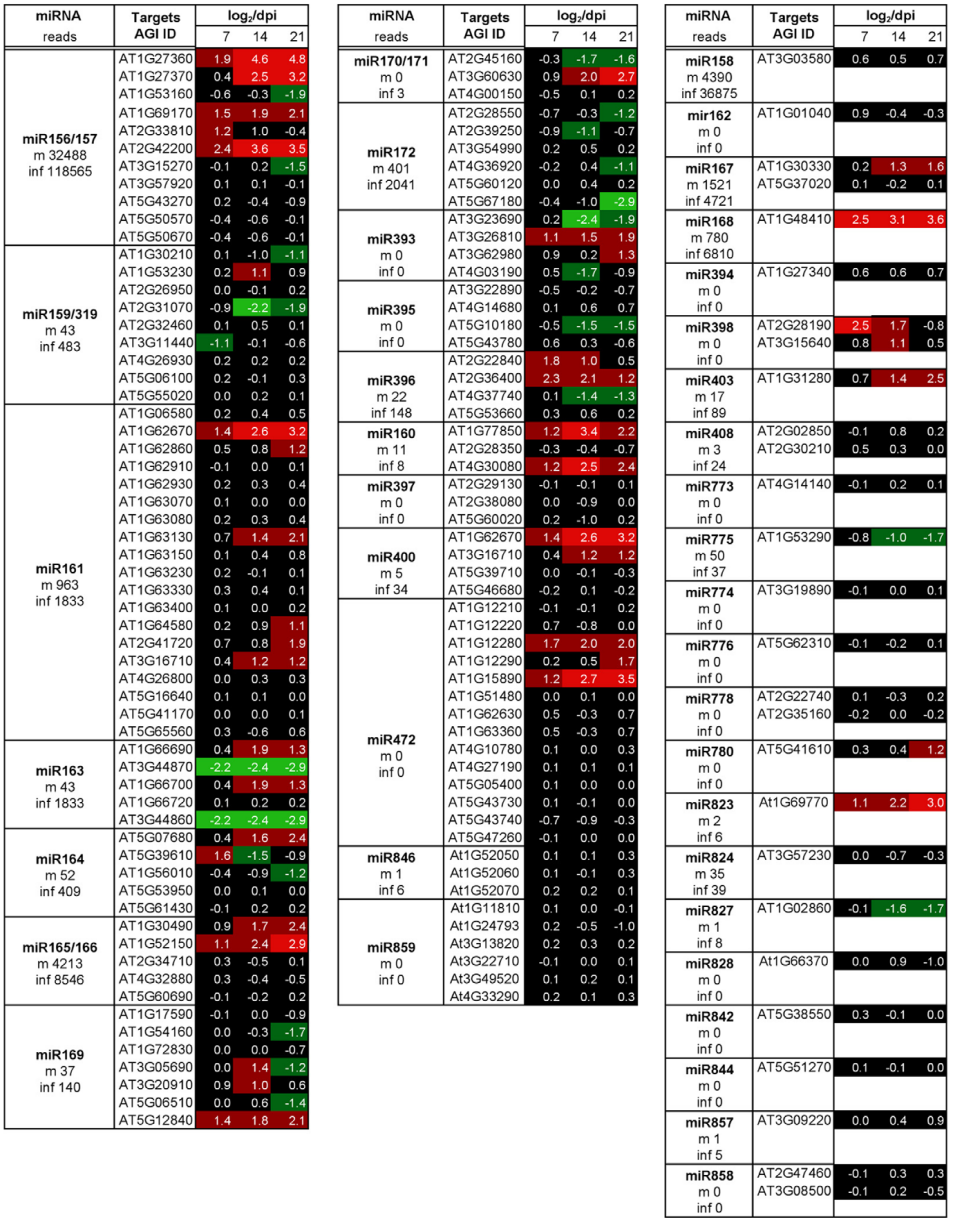
Changes in the level of miRNA target transcripts upon ORMV infection at 7, 14, and 21 dpi. Heatmap shows log_2_-fold change values for the mRNA targets of specific miRNAs. The miRNA reads in mock-treated (m) and ORMV-infected (inf) plants is shown. Although some miRNA targets show increased (red) and decreased (green) levels of expression upon infection, the majority of the miRNA target mRNAs does not show a change in the level of expression.

**Figure 7:**
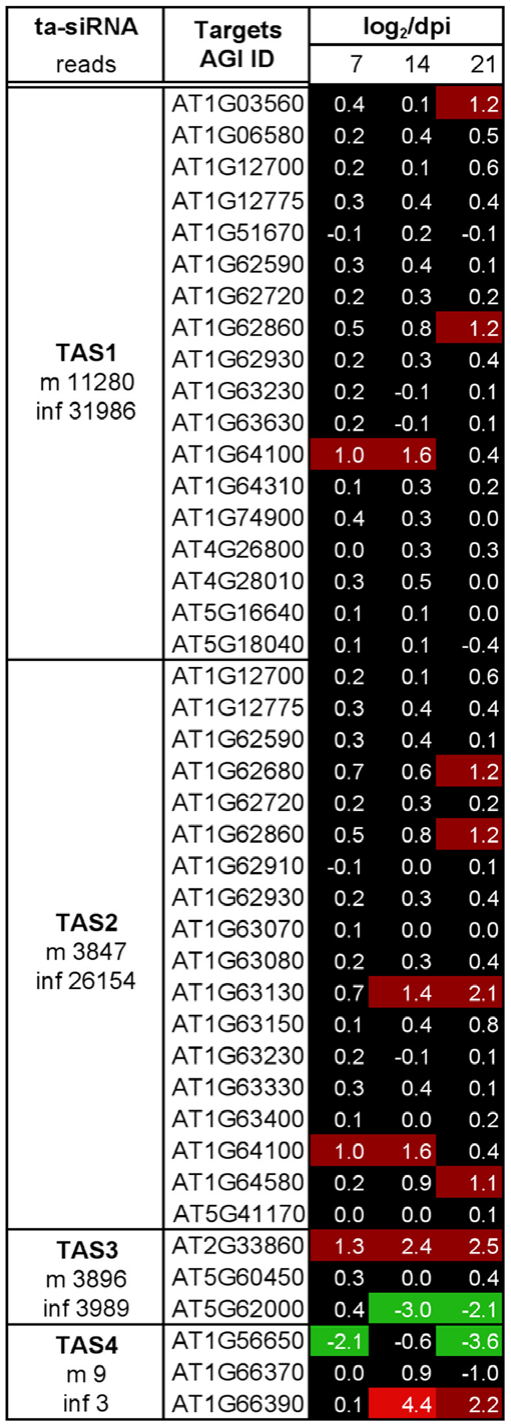
Changes in the level of ta-siRNA target transcripts upon ORMV infection at 7, 14, and 21 dpi. Heat map shows log_2_-fold change values for the mRNA targets of specific ta-siRNA classes. The majority of the ta-siRNA target mRNAs shows stable expression during infection.

We next addressed whether the strong increases in the levels of ta-siRNAs and miRNAs are correlated with similarly strong changes in the level of their mRNA targets. Although our transcriptome data revealed significant changes in the transcript levels of many genes (Figure 5), the levels of the majority of the miRNA and ta-siRNA target transcripts appeared rather stable (Figure 6 and Figure 7). The general down-regulation of targets expected if all over-accumulated miRNAs would engage in target cleavage was not observed. Rather, some of the targets show increases in their abundance. Examples are members of the SPL transcription factor family (targets of miR156/157), a member of the pentatricopeptide family (AT1G62670; target of miR161 and miR400), a GRF gene family transcription factor (AT2G36400, target of miR396), Auxin response factors (ARF) 16 and 17 (AT1G77850 and AT4G30080; targets of miR160), and genes encoding LRR disease resistance gene motifs (AT1G122280 and AT1G15890; targets of miR472). Consistent with previously published observations by others [25], [33], a strong increase was also found for AGO1 (AT1G48410), the target of miR168. Overall, stronger changes in miRNA and ta-siRNA target transcript levels are seen at later infection stages (21 dpi), whereas there are rather mild changes, if any, at the time point of sRNA analysis (7 dpi). Only two of the 248 tested miRNA and ta-siRNA target transcripts show reduced levels at all three time points, while 16 targets exhibit increased levels at all three time points. Twenty-one targets display increased expression levels at later time points (starting at 14 or at 21 dpi), and 20 targets show reduced expression levels at later time points (starting at 14 or at 21 dpi). Notably, changes in the levels of miRNAs targeting multiple genes (e.g. miR163, 164, 169, 171, 393, 156/157) did not trigger similar changes in all their known target transcripts. Rather, the targets belonging to groups controlled by the same miRNAs exhibit diverse changes. These observations indicate that the changes in miRNA and ta-siRNA levels in ORMV-infected cells do not lead to corresponding changes in the transcriptome.

### ORMV infection promotes expression of novel miRNA-like sRNAs from miRNA precursors

**Figure 8:**
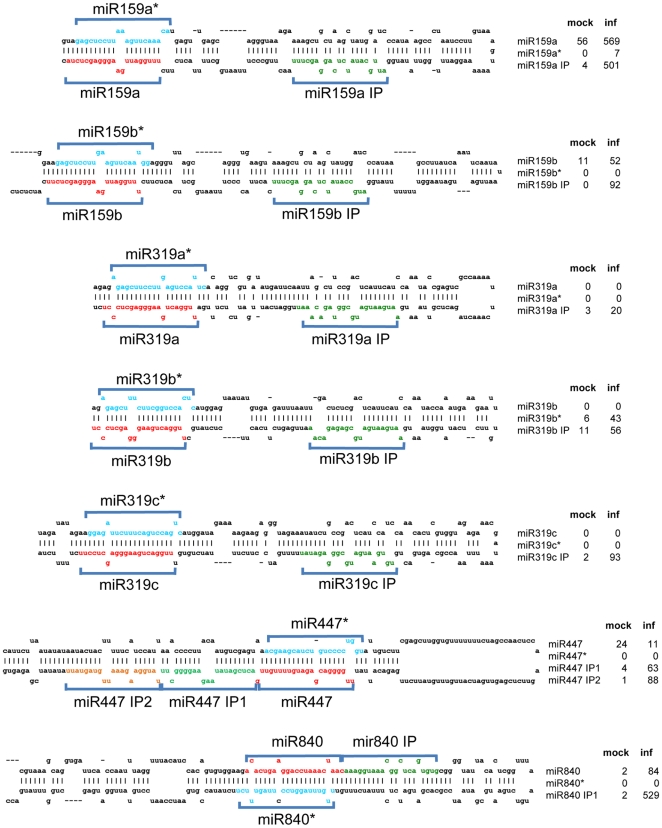
ml-siRNAs produced from miRNA precursor RNAs upon infection. The foldback structures of several miRNA precursor RNAs are shown. The sequences of mature miRNAs and miRNA* sequences are indicated in red and blue color, respectively. ml-siRNAs are depicted in green and yellow color. Since the ml-siRNAs are produced in phase (IP) with the miRNA and miRNA* sequences, they are designated according to the particular miRNA with the extension “IP” (for example, “miR159 IP”). The normalized reads (RPM) obtained for miRNAs, miRNA*, and ml-siRNA sequences for mock-treated (mock) and ORMV-infected plants (inf) are shown.

**Figure 9:**
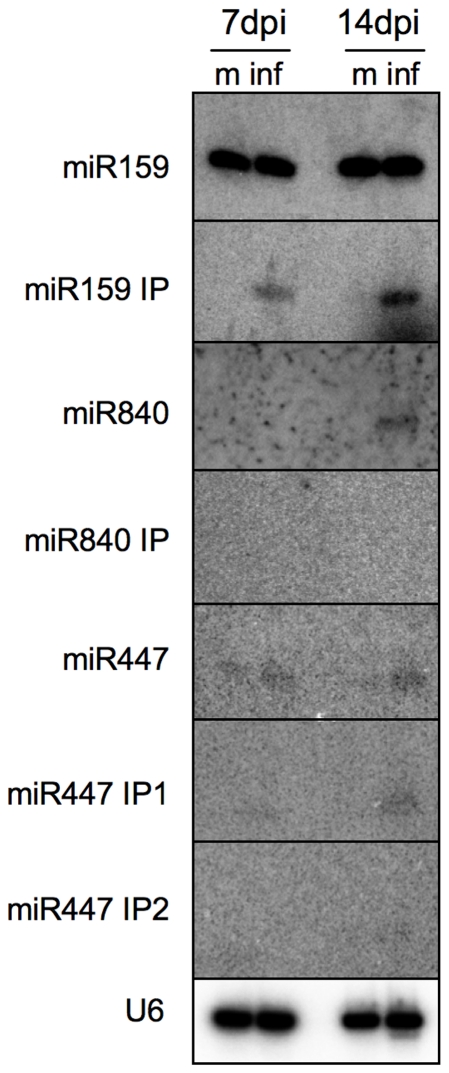
Northern blot analysis confirms the accumulation of ml-siRNAs in infected tissues. m, mock; inf, infected.

Our sequencing data revealed the presence of a substantial number of unique sRNAs derived from miRNA primary transcripts that do not represent the known miRNA or miRNA* sequences (Figure 8). Their unique accumulation upon virus infection was verified by sRNA blot hybridization (Figure 9). This finding indicates that miRNA precursors can generate multiple sRNAs including novel pathogen-inducible species. Apparently, these miRNA-like sRNAs (ml-sRNAs) are produced at very low levels under normal conditions whereas they are better processed or stabilized, and thus enriched, in plants challenged with ORMV. These ml-sRNAs are in phase with the canonical miRNAs, suggesting that they are produced during phased processing of the miRNA precursor RNAs by DCL1 [58]. Among the 19 ml-sRNAs identified in our experiment, eight were recently shown to be also induced by the bacterial pathogen *Pseudomonas* [36] (Table 2). Thus, accumulation of ml-sRNAs appears to be a common feature of bacterial and viral infection.

**Table 2:** MIR-encoded ml-siRNAs.

				Reads			
MIR	ml-siRNA	ml-siRNA sequence	size	m	inf	AGO^1^	PARE^2^
miR159A	159A_IP*	ATTGCATATCTCAGGAGCTTT	21	2	350	1,2,7	AT5G24620
miR159B	159B_IP*	ATGCCATATCTCAGGAGCTTT	21	0	64	1,2,7	
miR319A	319A_IP	AATGAATGATGCGGTAGACAA	21	2	14	1,2,4,5	
	319A_IP1	AATGAATGATGCGGTAGACAAA	22	0	2	1,2,4,5	
miR319B	319B_IP*	AATGAATGATGCGAGAGACAA	21	6	39	1,2	
miR319C	319C_IP	TGTGAATGATGCGGGAGATAT	21	1	65	1	
miR163	163_IP1	ATTATCCCCCGTGTTTTGTCC	21	0	3	1,2,4,5,7	AT4G11680
	163_IP2	CCAAAACCCGGTGGATAAAAT	21	0	5	1,4,5,7	AT3G52150
miR169F	169F_IP*	TGAAGGAATAACGAATGGAAT	21	2	0	1	
miR169L	169L_IP	TGGCGAAAAGAGTCATGTTTAA	22	1	6		
miR447	447_IP1*	ACTCGATATAAGAAGGGGCTT	21	2	44	1,2,4,5,7	
	447_IP2*	TATGGAAGAAATTGTAGTATT	21	1	61	1,2,4,5,7	
miR822	822_IP*	AAACAATATACGTTGCATCCC	21	2	15	1,2,4,7	
miR840	840_IP	AAAGGTAAACGGCTCAGTGTG	21	1	370		
miR841	841_IP1	CACATGCAACTCAAGACTAGA	21	10	1		AT1G01790
miR846	846_IP1	AATTGGATATGATAAATGGTA	21	0	8	2	AT4G38740, AT5G57520
	846_IP2*	AATTGGATATGATAAATGGTAA	22	1	9		
miR863	863_alter	ATGCGATTGAGAGCAACAAGACAT	24	158	207		
	863_alter	TGCGATTGAGAGCAACAAGAC	21	17	136	1,4	

*Present also in plants infected with *Pseudomonas syringae* (Zhang et al., 2010). **^1^**Potential association of ml-siRNA with specific AGO proteins. **^2^**Potential targets of ml-siRNA. Reads are RPM.

To determine whether enriched ml-sRNAs could play a role in RISC-mediated degradation of target transcripts, we used them as queries for a DegradomeSearch with StarBase (http://starbase.sysu.edu.cn/index.php), a public platform for exploring microRNA-target interaction maps from Argonaute CLIP-Seq (HITS-CLIP) and degradome sequencing (Degradome-Seq, PARE) data [59]. Using a penalty score of ≥4.5 and searching for targets indicated by ≥1 cleavage tags, we identified potential targets of 4 of the eleven newly identified ml-siRNAs in our database (miR163-IP1, miR163-IP2, miR841-IP1, and miR841-IP2). To further investigate the potential role of ml-siRNAs in target mRNA degradation we searched sequenced libraries of AGO-associated sRNAs [14], [19]. Indeed, here we found several indications of associations of ml-sRNAs with AGO proteins. Thus, miR163-IP1 was associated with AGO1, 2, 4, 5, and 7, miR163-IP2 was associated with AGO1, 4, 5, and 7, and miR846-IP1occurred in association with AGO2. These findings suggest that at least some of the virus-induced ml-sRNAs may function in the regulation of mRNA targets in association with specific AGO complexes. It will be interesting to determine the functions of these ml-siRNAs during infection and during normal plant development.

## Discussion

Plant-virus interaction triggers multiple plant defense pathways [60], including RNA silencing [21], [22]. To counteract RNA silencing, plant viruses encode diverse types of VSR that act at different steps in the silencing pathway [24]. Up to now, most of the investigated plant VSRs are pathogenic proteins [61], [62], [63]. Their activities contribute to the development of virus-induced disease symptoms by interfering with endogenous gene expression and endogenous sRNA pathways [25], [31], [64]. The small replicase subunit of tobamoviruses was identified as a pathogenicity determinant [65] and was shown to function as VSR [25], [26], [27], [66]. *In vitro* experiments indicate that this protein binds double-stranded sRNAs [25], [28], [30] and this binding has been proposed to account for the enrichment of both miRNAs and miRNA* sequences seen in infected plants [25], [26], [31]. A fraction of the accumulated sRNAs lacks methylation at the 3′ end, suggesting that replicase binding to sRNAs may interfere with their methylation by HEN1 [25], [26]. Point mutation in the methyltransferase domain of the replicase interferes with the silencing suppressing capacity of the protein, coupled with weakened pathogenic symptoms in plants and decreased accumulation of non-methylated miRNAs/siRNAs [26]. Although the ability of the replicase to bind sRNA duplexes *in vitro* may explain the enrichment for both miRNAs and miRNA* sequences seen in infected plants [25], [26], [31], it remained unclear whether the *in vivo*-enriched miRNAs and miRNA* sequences are indeed derived from replicase-stabilized duplexes or whether other virus-induced mechanisms play a role in sRNA enrichment. Moreover, it remained unclear whether the sRNA enrichments would impose changes in the host transcriptome. We have now gained critical insights into the tobamovirus interaction with RNA silencing, and sRNAs in particular, by characterizing the sRNA and transcriptome profiles of ORMV-infected *Arabidopsis* plants.

### ORMV infection causes global size-specific sRNA enrichment in systemically infected plants

Our deep sequencing data indicate that ORMV infection leads to a global enrichment of sRNA species that are predominantly 21 nt in length, including miRNAs and ta-siRNAs, other RDR6-dependent siRNAs, as well as siRNAs processed from inverted repeat loci. The observation that ORMV infection enriches 20–21 nt sRNAs but not 24 nt sRNAs is in agreement with an earlier study [29] and consistent with the observation made *in vitro* that the tobamoviral VSR protein binds sRNA duplexes in a size-specific manner [25]. Tagami et al (2007) found that infection by TMV-Cg leads to specific enrichment of miRNAs [31]. Our work now extends this conclusion by the demonstration that ORMV infection causes a general enrichment of 20–21 nt RNAs irrespective of their origin.

### Viral suppressor binding may not be the sole determinant of the enriched sRNA profile

The ability of the tobamoviral silencing suppressor to bind sRNA duplexes in a size-specific manner *in vitro* [25], [28], [30] has been proposed to explain the enrichment of miRNA* sequences along with their corresponding miRNAs observed *in vivo* [25], [26], [31]. However, our results rather suggest that additional factors play a role in the *in vivo* enrichment of sRNAs. In particular, we found that the enrichment of size-specific sRNAs is selective. Enriched ta-siRNAs and miRNA* sequences are characterized by a strong bias for those carrying a 5′ terminal guanine. Among ta-siRNAs, this bias is found for ta-siRNAs derived from TAS2 but not for those derived from other TAS genes. For miRNAs, we find this bias for the miRNA passenger strands rather than for the guide strands. The particular enrichment of miRNA* and TAS2-derived sequences with a 5′G cannot be explained by a bias in the population of sRNAs generated from the different precursors. Although the proportion of unique TAS ta-siRNAs with a 5′G is increased from 15% to 19% upon ORMV infection, their total number is increased from 25% to 69% of the total TAS2 ta-siRNA population (Table S2). The same is true for miRNA* sequences. Whereas the number of sequenced unique miRNA* sequences initiating with a 5′G is increased from 13 to 15 upon infection, their total number is increased 45 fold, i.e. from less than 500 reads to more than 20000 reads (Table S5). This represents a strong degree of enrichment of unique ta-siRNAs and miRNA* sequences starting with 5′G. Since RISC-associated AGO proteins were shown to preferentially associate and stabilize sRNAs according to their 5′ nucleotides [14], [18], [19], this finding suggests the involvement of specific AGO-associated effector complexes in the virus-induced sRNA enrichment. Interestingly, we found that the 5′nucleotide specific pattern of enrichment of ta-siRNAs (G>A = U>C) mimics that of vsRNAs (Figure S1, D). This may suggest that vsRNAs and ta-siRNAs are stabilized by similar complexes.

A role for host effector complexes in addition to the binding by the viral suppressor in the stabilization of sRNAs is also supported by the observation that ORMV infection causes a stronger increase in the levels of miRNA* sequences relative to that of the respective miRNAs. For example, miR160*, miR396*, and miR398*, are particularly highly over-represented as compared to their miRNA. This observation cannot solely be explained by the ability of the tobamoviral replicase to bind sRNA duplexes since this should lead to equal accumulation of both strands of the different duplexes. Another less likely possibility, which is in contradiction with earlier *in vitro* binding assays indicating that replicase does not bind single-stranded sRNAs [25], [28], is that the replicase binds only one of the two strands of sRNA duplexes and exhibits some kind of 5′ nucleotide preference similar to that of AGO proteins. However, we noted the enrichment of miRNA163 in infected plants. Given the preference for enrichment of 21 nt sRNAs, this 24 nt long miRNA may only be enriched when present in a duplex with its passenger strand, which forces the 24 nt miRNA to bulge out and thus to assume a length characteristic for 21 nt sRNA. This finding may suggest that the size selection for enrichment occurs at the level of duplexes. Thus, it may be conceivable that the ability of the viral suppressor to bind 21 nt sRNA duplexes plays a role in sRNA size selection whereas the final enrichment of single strands is caused by association with other proteins.

### Possible functions of enriched sRNAs during ORMV infection

Our observation that the enriched miRNA and ta-siRNA levels have no strong effects on the levels of their mRNA targets does not necessarily indicate that they are inactive. First, the targets of these sRNAs may be robustly regulated by feedback mechanisms (e.g. at the level of transcription) or may be controlled by established RISCs that are stable and thus resistant against virus-induced changes in sRNA levels. Second, since some of the mRNA targets show changes in expression levels, the virus-induced sRNA enrichment may occur predominantly in specific tissues in which the majority of the corresponding targets is not expressed. The enriched sRNAs may also function in sRNA-guided translational repression rather than in target transcript cleavage. Translational suppression by sRNA-guided AGO complexes is a common phenomenon in plants and animals [12], [67], [68]. For example, as we could confirm in parallel experiments (not shown), in Arabidopsis, accumulation of miR168 caused by distinct RNA viruses leads to AGO10-mediated translational inhibition rather than to AGO1-mediated cleavage of the AGO1 mRNA [33]. Moreover, recent reports suggest that plant miRNAs can also mediate DNA methylation [11], [69], which adds yet another dimension by which the enriched sRNAs may act.

With regard to functional diversification it is also important to note that a large proportion of the sRNAs that accumulate in tobamovirus-infected plants is not methylated [25], [26], [29]. Although non-methylated sRNAs are usually degraded [70], [71], they may be stabilized upon association with specific effectors. A precedent for this hypothesis is provided by miRNAs in Drosophila that bind to either AGO1 or AGO2 proteins, dependent on whether they are methylated at the 3′ end by HEN1, or not [72], [73], [74]. Moreover, unlike the methylated sRNAs associated with AGO2, the AGO1-bound non-methylated sRNAs undergo target-guided tailoring and trimming, which contributes to the efficiency of sRNAs with limited complementarity to the target [75]. Thus, the enrichment of size- and nucleotide-specific Arabidopsis sRNAs that are not methylated and thus potentially changed at their 3′ end may represent an important mechanism by which ORMV and potentially other viruses could diversify sRNA function during infection. Our analysis revealed the particular enrichment of sRNAs with 5′ terminal guanine. Notably, it is not known which of the AGO proteins is predominantly associated with 21 nt 5′G-sRNAs. It is also possible that non-AGO proteins may functionally associate with 5′G-sRNAs and other enriched sRNAs to form effector complexes with potentially diverse and yet unknown functions.

The enrichment of miRNA*s suggests that they might have a conditional function, as may also be supported by their association with predicted targets and the presence of potential target RNA cleavage products in degradome databases [76].

### miRNA precursors produce novel sRNAs in response to pathogen infection

In addition to miRNA* sequences, virus infection also causes the accumulation of ml-sRNAs, which also may play important roles during infection. The detection of ml-sRNAs indicates that miRNA precursors can produce additional sRNAs in Arabidopsis upon virus infection. Processing of multiple miRNAs from one precursor is a common phenomenon in animals where miRNA precursors often fold into complex secondary structures with multiple hairpins, each coding for one or more miRNAs. In plants, miRNA precursors usually form one hairpin coding for one miRNA. However, in rare cases, plant miRNA precursors can code for more than one miRNA. In these cases, miRNAs were shown to be processed sequentially from the longer hairpins by DCL1 from the hairpin base [58]. In rice, in addition to DCL1, other DCLs are also involved in processing multiple miRNAs from one-hairpin precursors [77].

The ml-sRNAs that we have identified here are rare or absent in non-infected tissues and accumulate to high levels upon ORMV infection. We identified 19 ml-siRNAs derived from 14 precursors (Table 2). The enrichment and identification of ml-siRNAs emphasizes the advantage of virus infection for the identification of novel sRNAs [31]. Interestingly, a significant number of different ml-siRNAs is also generated in Arabidopsis plants infected with *Pseudomonas syringae* [36] suggesting that the synthesis or stabilization of ml-siRNAs respond to common factors triggered by infection with bacterial or viral pathogens. Sequences with homology to ml-siRNAs occur in sRNA databases of a wide range of organisms indicating that they play important roles and have been conserved during evolution [36]. Similar to Zhang and colleagues (2010), we found ml-siRNAs arranged in phase with miRNAs and miRNA*s. The ml-siRNAs of miR159 and miR319 precursors are located towards the loop of the precursors, separated by one phase from the miRNA sequence at the lower stems. Since miR159 and miR319 are generated by sequential DCL cleavage of the precursors starting at the loop [78], [79], the ml-siRNAs are likely generated during normal miRNA processing. However, whereas the miR159/319 ml-siRNAs may be unstable under normal conditions, they may be stabilized in virus-infected tissues. A loop-based processing mechanism may also apply to the precursors of miR840 and miR846, since also here the ml-siRNAs identified in our data are located towards the loop. We also found other cases, for example pre-miR447, where two ml-siRNAs located near the hairpin base are processed from phases directly adjacent to the miRNA located next to the loop. This situation is consistent with the canonical base-to-loop processing mode, where miRNA processing is initiated by a cut close to the base of the stem [80]. Although the same applies to ml-siRNAs of the miR169 and miR822 precursors according to our data, Zhang et al. (2010) found ml-siRNAs located on either side of miR822. Whether the ml-siRNAs and miRNAs of the stem-based pathway may originate by consecutive, in-phase cleavage as in the case of the miR159/319 precursors or whether these sRNAs are produced via independent cleavage and release from different precursor molecules remains to be seen. Although the number of unique ml-siRNAs found by Zhang et al (2010) is higher, we found ml-siRNAs derived from miR840 and miR319c precursors, which were not detected in the previous study. Further studies are needed to determine whether this difference is specific to stimuli (e.g. infection by bacteria versus virus) or whether this only reflects the efficiency by which unique sRNAs are sequenced. It appears likely that the ml-siRNAs accumulating in virus-infected tissues are caused by stabilization by the VSR or by another virus-induced effector complex. Bacteria were recently shown to encode silencing suppressors [81]. Thus, ml-siRNAs may represent potential anti-pathogen agents triggered by both viral and bacterial pathogens.

## Materials and Methods

### Plant materials and virus infection

*Arabidopsis thaliana col-0* plants were grown in humidity-controlled growth chambers at 21°C using a 12 h/12 h light/dark cycle. *Nicotiana benthamiana* plants were grown under greenhouse conditions at 24°C using a 16 h/8 h light/dark cycle. To generate the inoculum for ORMV infection, *N. benthamiana* plants were inoculated with ORMV RNA *in vitro* transcribed from an infectious clone. Crude, virus-containing extracts (sap) from these infected plants as well as virus-free extracts from non-infected plants were used for mechanical inoculation of young Arabidopsis plants (5 leaves stage). The rosette leaves were harvested at 7 dpi, 14 dpi, and 21 dpi. The harvested leaves of 16–20 mock- or ORMV-inoculated plants were pooled per sample. Three independent samples of each treatment and time point were prepared.

### Sample preparation for RNA profiling and deep sequencing

Total RNA extracts were prepared by using classical Trizol (*Invitrogen*, Switzerland) extraction protocols following the instructions of the producer. The quality of the samples was verified by hybridization with specific probes to detect viral RNA and viral/endogenous sRNAs. Affimetrix gene chip hybridization for RNA profiling was performed at the Functional Genomics Center Zurich. The resulting Affimetrix microarray dataset was analyzed by using the R environment (http://www.R-project.org) [82] and Bioconductor software (http://www.biocinductor.org). For sRNA analysis, the total RNA samples were separated by gel electrophoresis. 18–30 nt sRNAs were isolated form the gel, ligated with adapters and sequenced using Solexa technology (ServiceXS B.V., Leiden, Netherlands; http://www.servicexs.com/).

### Processing of deep sequencing data

sRNA libraries were sequenced on an Illumina Genome Analyzer using the 36-cycle Solexa Sequencing Kit. The Illumina Gerald pipeline was used to process and extract the first 36 bases of the runs and a total of 6,658,605 raw sequence tags (3,447,032 reads for mock-inoculated samples and 3,211,573 reads for virus-inoculated samples) were generated. Following removal of adapter sequences the reads were grouped and counted according to sequence identity using a customized Python script (available upon request). The reads were mapped against the ORMV and *A. thaliana* genomes using Bowtie software [83]. All read counts were normalized to adjust for differences in library size and coverage to reads per million (RPM) according to the total read count in each library. Thus, each raw read count is multiplied by 10^6^ and then divided by the total read count of the whole library. This normalization step allows for direct comparisons between the data sets.

### Verification of sRNA and mRNA levels

sRNAs were detected by Northern blot hybridization using radiolabeled oligonucleotide probes as previously described [84]. The quality of RNA profiling and the infection-induced response at the level of transcripts were verified by quantifying several transcripts by qRT-PCR.

### Target prediction and PARE database mining

sRNA targets were identified by searching degradome databases with the StarBase on-line tool [59], using a penalty score ≥4.5 and ≥1 cleavage tags.

Potential associations of sRNAs with AGO proteins were identified by searching databases of AGO-associated sRNAs. The GEO (http://www.ncbi.nlm.nih.gov/geo/) datasets used in the assay were GSM253622 (AGO1), GSM253623 and GSM304285 (AGO2), GSM253624 (AGO4), GSM253625 (AGO5), and GSM304283 (AGO7).

## Supporting Information

Figure S1 **The viral and endogenous sRNA profile. (A)** Number of normalized sRNA reads (RPM) mapped to the *Arabidopsis thaliana* (A.t.) and viral genomes. **(B)** Proportion of virus- and plant-derived sRNA reads in the population of sequenced and mapped sRNAs of ORMV-infected plants. **(C)** Size distribution of vsRNAs. Virus infection increases the number of 21 nt sRNAs whereas the number of 24 nt sRNAs is reduced. **(D)** The normalized frequency (RPM) of vsRNAs according to their specific 5′ nucleotide. **(E)** vsRNAs mapped to the plus strand (black) and minus strand (grey) of the ORMV genome.
(TIF)

Table S1 **Size-specific profile of ta-siRNAs in mock- and ORMV-treated plants (7 dpi).**
(DOC)

Table S2 **5**′**nucleotide-specific accumulation of TAS2-derived 21** **nt siRNAs in ORMV-infected tissue (7 dpi).**
(DOC)

Table S3 **Size-specific profile of siRNAs encoded by RDR6-dependent loci in mock- and ORMV-treated plants.**
(DOC)

Table S4 **Size-specific profile of siRNAs encoded by IR71 in mock- and ORMV-treated plants (7 dpi).**
(DOC)

Table S5 miRNA and miRNA* reads in mock and ORMV-infected plants.
(DOC)

Table S6 **Functional annotations for genes with modulated expression (log_2_-fold change, p<0.001) upon ORMV infection.**
(DOC)
