# Supplementary figures and images for: Specific Impact of Tobamovirus Infection on the Arabidopsis Small RNA Profile
Source: PLoS One. 2011 May 10;6(5):e19549. doi: 10.1371/journal.pone.0019549 (PMC3091872; doi:10.1371/journal.pone.0019549)

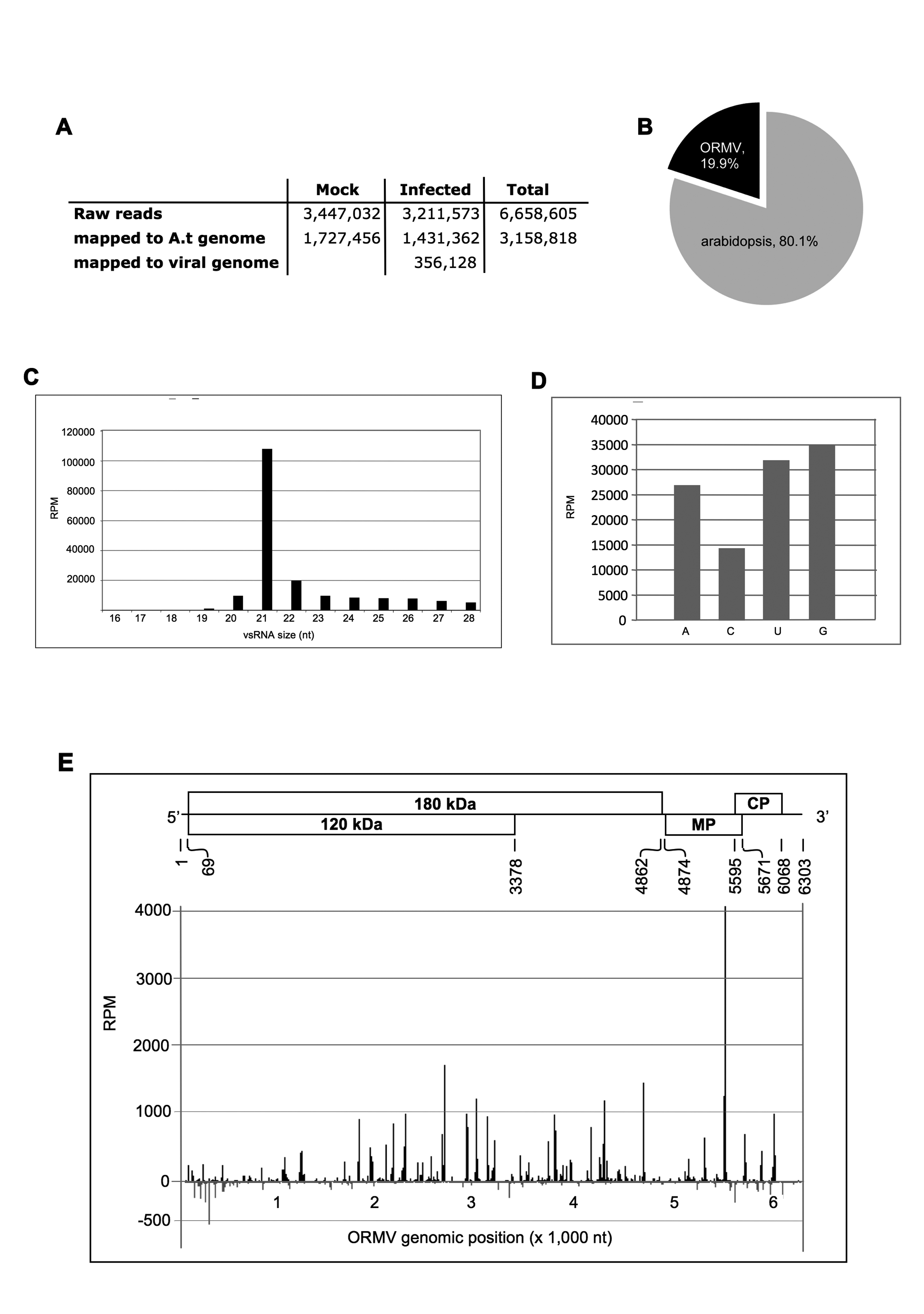

Supplement: Figure S1 — The viral and endogenous sRNA profile. (A) Number of normalized sRNA reads (RPM) mapped to the Arabidopsis thaliana (A.t.) and viral genomes. (B) Proportion of virus- and plant-derived sRNA reads in the population of sequenced and mapped sRNAs of ORMV-infected plants. (C) Size distribution of vsRNAs. Virus infection increases the number of 21 nt sRNAs whereas the number of 24 nt sRNAs is reduced. (D) The normalized frequency (RPM) of vsRNAs according to their specific 5′ nucleotide. (E) vsRNAs mapped to the plus strand (black) and minus strand (grey) of the ORMV genome. (TIF) [file pone.0019549.s001.tif]
