# Supplementary material for: Specific Impact of Tobamovirus Infection on the Arabidopsis Small RNA Profile
Source: PLoS One. 2011 May 10;6(5):e19549. doi: 10.1371/journal.pone.0019549 (PMC3091872; doi:10.1371/journal.pone.0019549)
Supplement: Table S1 — Size-specific profile of ta-siRNAs in mock- and ORMV-treated plants (7 dpi). (DOC) [file pone.0019549.s002.doc]

Table S1. Size-specific profile of ta-siRNAs in mock- and ORMV-treated plants (7dpi)

|  |  | **20nt** | | **21nt** | | **22nt** | | **23nt** | | **24nt** | | **Total reads** | |  |
| --- | --- | --- | --- | --- | --- | --- | --- | --- | --- | --- | --- | --- | --- | --- |
| **Gene** |  | m | inf | m | inf | m | inf | m | inf | m | inf | m | inf | inf/m |
| **TAS1a** | U | 58 | 115 | 91 | 203 | 93 | 114 | 63 | 66 | 100 | 89 | 405 | 587 | 1.45 |
|  | T | 255 | 1851 | 3791 | 18234 | 1306 | 1236 | 234 | 274 | 928 | 732 | 6514 | 22327 | 3.43 |
| **TAS1b** | U | 20 | 65 | 38 | 51 | 41 | 52 | 21 | 15 | 34 | 33 | 154 | 215 | 1.4 |
|  | T | 32 | 668 | 244 | 3308 | 103 | 144 | 40 | 37 | 173 | 91 | 592 | 4248 | 7.18 |
| **TAS1c** | U | 66 | 179 | 105 | 253 | 87 | 118 | 57 | 73 | 121 | 58 | 436 | 680 | 1.56 |
|  | T | 175 | 682 | 930 | 8133 | 958 | 1495 | 496 | 576 | 2548 | 2877 | 5107 | 13763 | 2.69 |
| **TAS2** | U | 43 | 135 | 85 | 179 | 71 | 103 | 45 | 50 | 56 | 52 | 300 | 518 | 1.73 |
|  | T | 155 | 1195 | 523 | 14607 | 949 | 1407 | 196 | 219 | 279 | 217 | 2102 | 17645 | 8.39 |
| **TAS3a** | U | 34 | 40 | 58 | 110 | 45 | 54 | 30 | 34 | 64 | 57 | 232 | 294 | 1.27 |
|  | T | 67 | 92 | 660 | 1726 | 742 | 494 | 91 | 69 | 583 | 312 | 2143 | 2694 | 1.26 |
| **TAS3b** | U | 5 | 4 | 8 | 7 | 6 | 4 | 14 | 4 | 16 | 17 | 49 | 36 | 0.75 |
|  | T | 14 | 13 | 10 | 9 | 24 | 14 | 34 | 4 | 47 | 41 | 130 | 81 | 0.62 |
| **TAS3c** | U | 0 | 1 | 0 | 3 | 0 | 0 | 1 | 1 | 0 | 0 | 1 | 5 | 8.45 |
|  | T | 0 | 1 | 0 | 6 | 0 | 0 | 1 | 1 | 0 | 0 | 1 | 8 | 13.3 |
| **TAS4** | U | 0 | 2 | 2 | 0 | 1 | 0 | 0 | 0 | 0 | 0 | 3 | 2 | 0.72 |
|  | T | 0 | 2 | 5 | 0 | 1 | 0 | 0 | 0 | 0 | 0 | 5 | 2 | 0.4 |
| **sum** | U | 226 | 541 | 388 | 806 | 344 | 444 | 230 | 242 | 391 | 305 | 1579 | 2338 | 1.48 |
|  | T | 698 | 4504 | 6163 | 46023 | 4082 | 4790 | 1092 | 1180 | 4558 | 4270 | 16593 | 60767 | 3.66 |
| **T/U** |  | 3.08 | 8.33 | 15.89 | 57.13 | 11.9 | 10.8 | 4.75 | 4.87 | 11.7 | 14 | 10.51 | 25.99 | 2.47 |
| **FC** |  | 6.46 | | 7.468 | | 1.17 | | 1.08 | | 0.94 | | 3.662 | |  |
| **FCTU** |  | 2.7 | | 3.596 | | 0.91 | | 1.02 | | 1.2 | | 2.472 | |  |

U, unique reads; T, total reads; FC, fold change of T; FCTU, fold change of T/U; m, mock-inoculated; inf, ORMV-infected. Reads are RPM.
