# Supplementary material for: Specific Impact of Tobamovirus Infection on the Arabidopsis Small RNA Profile
Source: PLoS One. 2011 May 10;6(5):e19549. doi: 10.1371/journal.pone.0019549 (PMC3091872; doi:10.1371/journal.pone.0019549)
Supplement: Table S2 — 5′nucleotide-specific accumulation of TAS2-derived 21 nt siRNAs in ORMV-infected tissue (7 dpi). (DOC) [file pone.0019549.s003.doc]

**Table S2. 5’nucleotide-specific accumulation of TAS2-derived 21 nt siRNAs in ORMV-infected tissue (7dpi)**

|  | **m** | | **inf** | |
| --- | --- | --- | --- | --- |
|  | **U** | **T** | **U** | **T** |
| **A** | 36 | 177 | 70 | 2110 |
| **C** | 9 | 52 | 18 | 211 |
| **G** | 14 | 151 | 38 | 10398 |
| **U** | 34 | 213 | 76 | 2410 |
| **Total** | 94 | 593 | 202 | 15129 |
| **%G** | **15** | **25** | **19** | **69** |

m, mock-inoculated; inf, ORMV-infected. Reads are RPM.
