# Supplementary material for: Specific Impact of Tobamovirus Infection on the Arabidopsis Small RNA Profile
Source: PLoS One. 2011 May 10;6(5):e19549. doi: 10.1371/journal.pone.0019549 (PMC3091872; doi:10.1371/journal.pone.0019549)
Supplement: Table S3 — Size-specific profile of siRNAs encoded by RDR6-dependent loci in mock- and ORMV-treated plants. (DOC) [file pone.0019549.s004.doc]

**Table S3. Size-specific profile of siRNAs encoded by RDR6-dependent loci in mock- and ORMV-treated plants**

|  |  | **20nt** | | **21nt** | | **22nt** | | **23nt** | | **24nt** | | **total reads** | |  |
| --- | --- | --- | --- | --- | --- | --- | --- | --- | --- | --- | --- | --- | --- | --- |
| **Gene** |  | m | inf | m | inf | m | inf | m | inf | m | inf | m | inf | inf/m |
| **At1g63080** | U | 1 | 8 | 29 | 110 | 34 | 31 | 9 | 3 | 8 | 2 | 80 | 155 | 1.93 |
|  | T | 1 | 11 | 92 | 322 | 94 | 56 | 11 | 5 | 9 | 4 | 207 | 398 | 1.92 |
| **At1g62910** | U | 1 | 13 | 43 | 130 | 37 | 41 | 6 | 9 | 17 | 15 | 104 | 207 | 1.98 |
|  | T | 3 | 28 | 374 | 1383 | 150 | 121 | 9 | 12 | 68 | 64 | 604 | 1608 | 2.66 |
| **At1g63330** | U | 2 | 3 | 8 | 39 | 8 | 9 | 2 | 2 | 3 | 3 | 23 | 57 | 2.54 |
|  | T | 9 | 72 | 437 | 1378 | 12 | 17 | 2 | 7 | 5 | 3 | 466 | 1478 | 3.17 |
| **At1g63070** | U | 1 | 2 | 1 | 11 | 4 | 8 | 1 | 1 | 3 | 2 | 10 | 24 | 2.41 |
|  | T | 1 | 10 | 1 | 22 | 13 | 21 | 1 | 1 | 3 | 2 | 20 | 57 | 2.88 |
| **At1g63150** | U | 2 | 9 | 21 | 73 | 20 | 20 | 3 | 6 | 12 | 14 | 57 | 122 | 2.12 |
|  | T | 3 | 41 | 183 | 604 | 53 | 36 | 8 | 13 | 77 | 82 | 324 | 777 | 2.40 |
| **At1g12820** | U | 1 | 3 | 8 | 25 | 8 | 7 | 4 | 1 | 5 | 6 | 25 | 43 | 1.67 |
|  | T | 1 | 5 | 47 | 103 | 23 | 17 | 5 | 1 | 11 | 8 | 87 | 134 | 1.54 |
| **At1g62590** | U | 0 | 1 | 5 | 23 | 4 | 4 | 1 | 0 | 2 | 1 | 12 | 29 | 2.41 |
|  | T | 0 | 1 | 8 | 33 | 5 | 4 | 1 | 0 | 2 | 1 | 16 | 39 | 2.50 |
| **At1g62930** | U | 2 | 12 | 25 | 81 | 29 | 33 | 6 | 6 | 11 | 8 | 73 | 140 | 1.92 |
|  | T | 2 | 24 | 75 | 374 | 127 | 92 | 8 | 6 | 17 | 8 | 228 | 504 | 2.22 |
| **At1g63130** | U | 1 | 15 | 42 | 103 | 35 | 44 | 9 | 13 | 19 | 16 | 105 | 190 | 1.80 |
|  | T | 1 | 20 | 560 | 1833 | 167 | 124 | 17 | 20 | 49 | 38 | 793 | 2036 | 2.57 |
| **At5g41610** | U | 0 | 0 | 0 | 1 | 0 | 0 | 0 | 0 | 0 | 0 | 0 | 1 |  |
|  | T | 0 | 0 | 0 | 1 | 0 | 0 | 0 | 0 | 0 | 0 | 0 | 1 |  |
| **At5g38850** | U | 2 | 28 | 79 | 259 | 64 | 80 | 12 | 6 | 16 | 11 | 173 | 384 | 2.23 |
|  | T | 2 | 39 | 186 | 879 | 148 | 124 | 13 | 6 | 18 | 14 | 366 | 1062 | 2.90 |
| **At1g63400** | U | 1 | 5 | 9 | 44 | 13 | 17 | 2 | 5 | 9 | 8 | 33 | 80 | 2.41 |
|  | T | 2 | 11 | 92 | 207 | 168 | 125 | 2 | 5 | 21 | 20 | 286 | 369 | 1.29 |
| **sum** | U | 12 | 99 | 268 | 899 | 256 | 293 | 57 | 52 | 103 | 87 | 696 | 1431 | 2.06 |
|  | T | 25 | 262 | 2054 | 7139 | 961 | 738 | 77 | 77 | 279 | 247 | 3396 | 8462 | 2.49 |
| **T/U** |  | 2.0 | 2.6 | 7.7 | 7.9 | 3.8 | 2.5 | 1.4 | 1.5 | 2.7 | 2.8 | 4.9 | 5.9 |  |
| **FC** |  | 10.52 | | 3.47 | | 0.77 | | 1.00 | | 0.88 | | 2.49 | |  |
| **FCTU** |  | 1.29 | | 1.04 | | 0.67 | | 1.08 | | 1.05 | | 1.21 | |  |

U, unique reads; T, total reads; FC, fold change of T; FCTU, fold change of T/U; m, mock-inoculated; inf, ORMV-infected. Reads are RPM.
