# Supplementary material for: Specific Impact of Tobamovirus Infection on the Arabidopsis Small RNA Profile
Source: PLoS One. 2011 May 10;6(5):e19549. doi: 10.1371/journal.pone.0019549 (PMC3091872; doi:10.1371/journal.pone.0019549)
Supplement: Table S4 — Size-specific profile of siRNAs encoded by IR71 in mock- and ORMV-treated plants (7 dpi). (DOC) [file pone.0019549.s005.doc]

**Table S4. Size-specific profile of siRNAs encoded by IR71 in mock- and ORMV-treated plants (7dpi)**

|  |  | **24nt** | | **23nt** | | **22nt** | | **21nt** | | **20nt** | | **Total Reads** | |
| --- | --- | --- | --- | --- | --- | --- | --- | --- | --- | --- | --- | --- | --- |
| **Gene** |  | m | inf | m | inf | m | inf | m | inf | m | inf | m | inf |
| **IR71** | U | 864 | 844 | 311 | 290 | 668 | 993 | 223 | 762 | 41 | 118 | 3639 | 4304 |
|  | T | 9172 | 8330 | 1305 | 1495 | 12275 | 19361 | 902 | 5994 | 58 | 266 | 40963 | 50735 |
| **T/U** |  | 10.6 | 9.9 | 4.2 | 5.2 | 18.4 | 19.5 | 4.0 | 7.9 | 1.4 | 2.3 | 11.3 | 11.8 |
| **FC** |  | 0.9 | | 1.1 | | 1.6 | | 6.6 | | 4.6 | | 1.2 | |
| **FCTU** |  | 0.9 | | 1.2 | | 1.1 | | 1.9 | | 1.6 | | 1.0 | |

U, unique reads; T, total reads; FC, fold change of T; FCTU, fold change of T/U; m, mock-inoculated; inf, ORMV-infected. Reads are RPM.
