# Supplementary material for: Specific Impact of Tobamovirus Infection on the Arabidopsis Small RNA Profile
Source: PLoS One. 2011 May 10;6(5):e19549. doi: 10.1371/journal.pone.0019549 (PMC3091872; doi:10.1371/journal.pone.0019549)
Supplement: Table S5 — miRNA and miRNA* reads in mock and ORMV-infected plants. (DOC) [file pone.0019549.s006.doc]

**Table S5. miRNA and miRNA* reads in mock and ORMV-infected plants**

|  | **miRNA** | | | | **miRNA*** | | | |
| --- | --- | --- | --- | --- | --- | --- | --- | --- |
| **miRNA** | **5’N** | **m** | **inf** | **FC** | **5’N** | **m** | **inf** | **FC** |
| miR156 | U | 7253 | 40431 | 5.6 | G | 46 | 8 | 0.2 |
| miR157 | U | 25234 | 78135 | 3.1 | G | 38 | 146 | 3.8 |
| miR158 | U | 4390 | 36875 | 8.4 | C | 1 | 163 | 141.2 |
| miR159 | U | 43 | 483 | 11.3 | G | 0 | 7 |  |
| miR160 | U | 11 | 8 | 0.8 | G | 50 | 2391 | 47.5 |
| miR161 | U | 963 | 1833 | 1.9 | - | 0 | 0 |  |
| miR162 | U | 0 | 0 |  | G | 19 | 439 | 23.0 |
| miR163 | U | 43 | 409 | 9.4 | U | 0 | 0 |  |
| miR164 | U | 52 | 291 | 5.7 | C | 0 | 12 |  |
| miR165 | U | 391 | 1051 | 2.7 | G | 24 | 699 | 28.7 |
| miR166 | U | 3822 | 7494 | 2.0 | G | 20 | 919 | 45.3 |
| miR167 | U | 1521 | 4721 | 3.1 | G | 5 | 155 | 29.8 |
| miR168 | U | 760 | 6810 | 9.0 | C | 12 | 434 | 35.7 |
| miR169 | C | 37 | 140 | 3.8 | G | 3 | 46 | 15.9 |
| miR170 | U | 0 | 0 |  | U | 0 | 10 |  |
| miR171 | U | 0 | 3 |  | U | 11 | 523 | 47.6 |
| miR172 | A | 401 | 2041 | 5.1 | G | 1 | 20 | 35.0 |
| miR173 | U | 1237 | 2154 | 1.7 | G | 4 | 203 | 50.0 |
| miR1886 | U | 0 | 2 |  | - | 0 | 0 |  |
| miR1888 | U | 8 | 15 | 2.0 | - | 0 | 0 |  |
| miR319 | U | 0 | 0 |  | A | 3 | 30 | 8.6 |
| miR390 | A | 45 | 959 | 21.2 | C | 0 | 3 |  |
| miR391 | U | 76 | 430 | 5.6 | A | 32 | 99 | 3.1 |
| miR396 | U | 22 | 148 | 6.7 | G | 173 | 6865 | 39.7 |
| miR398 | U | 0 | 0 |  | G | 72 | 8713 | 120.4 |
| miR399 | U | 0 | 2 |  | G | 1 | 8 | 13.3 |
| miR400 | U | 5 | 34 | 6.6 | G | 0 | 0 |  |
| miR403 | U | 17 | 89 | 5.1 | U | 0 | 0 |  |
| miR406 | U | 0 | 0 |  | U | 0 | 0 |  |
| miR408 | A | 3 | 24 | 7.0 | C | 337 | 3799 | 11.3 |
| miR447 | U | 22 | 27 | 1.2 | A | 0 | 0 |  |
| miR472 | U | 0 | 0 |  | U | 68 | 5450 | 79.8 |
| miR773 | U | 0 | 0 |  | G | 0 | 26 |  |
| miR775 | U | 50 | 37 | 0.7 | C | 2 | 17 | 10.1 |
| miR777 | U | 1 | 1 | 1.2 | G | 0 | 0 |  |
| miR779 | U | 1 | 57 | 97.8 | - | 0 | 0 |  |
| miR781 | U | 0 | 0 |  | A | 1 | 7 | 12.1 |
| miR783 | A | 0 | 0 |  | A | 6 | 13 | 2.3 |
| miR822 | U | 24 | 211 | 8.7 | U | 0 | 0 |  |
| miR823 | U | 2 | 6 | 3.2 | C | 0 | 0 |  |
| miR824 | U | 35 | 39 | 1.1 | C | 1 | 6 | 4.8 |
| miR825 | U | 8 | 115 | 14.1 | U | 0 | 0 |  |
| miR827 | U | 1 | 8 | 7.2 | U | 0 | 0 |  |
| miR829 | A | 0 | 0 |  | - | 0 | 0 |  |
| miR830 | U | 0 | 3 |  | U | 0 | 0 |  |
| miR833 | U | 3 | 7 | 2.4 | - | 0 | 0 |  |
| miR835 | U | 3 | 17 | 5.0 | - | 0 | 0 |  |
| miR837 | A | 0 | 0 |  | - | 2 | 10 | 4.5 |
| miR840 | A | 1 | 100 | 86.3 | U | 0 | 0 |  |
| miR841 | U | 0 | 0 |  | C | 0 | 6 |  |
| miR843 | U | 2 | 8 | 4.8 | C | 0 | 0 |  |
| miR844 | U | 0 | 0 |  | U | 0 | 0 |  |
| miR846 | U | 1 | 6 | 4.8 | U | 0 | 0 |  |
| miR847 | U | 0 | 0 |  | U | 0 | 0 |  |
| miR848 | U | 28 | 45 | 1.6 | G | 0 | 0 |  |
| miR850 | U | 21 | 318 | 15.3 | U | 2 | 48 | 27.8 |
| miR851 | U | 0 | 0 |  | - | 0 | 9 |  |
| miR852 | A | 3 | 5 | 1.4 | G | 0 | 0 |  |
| miR854 | G | 1 | 3 | 4.8 | U | 0 | 0 |  |
| miR857 | U | 1 | 5 | 8.4 | A | 0 | 0 |  |
| miR859 | U | 0 | 0 |  | U | 1 | 12 | 20.5 |
| miR860 | U | 0 | 1 |  | A | 0 | 0 |  |
| miR862 | A | 0 | 4 |  | - | 0 | 0 |  |
| miR863 | U | 0 | 0 |  | - | 0 | 0 |  |
| miR864 | U | 0 | 2 |  | - | 0 | 0 |  |
| miR865 | U | 0 | 0 |  | - | 0 | 0 |  |

5’N, 5’ nucleotide; FC, fold change; m, mock-inoculated; inf, ORMV-infected. Reads are RPM.
