# Supplementary material for: Specific Impact of Tobamovirus Infection on the Arabidopsis Small RNA Profile
Source: PLoS One. 2011 May 10;6(5):e19549. doi: 10.1371/journal.pone.0019549 (PMC3091872; doi:10.1371/journal.pone.0019549)
Supplement: Table S6 — Functional annotations for genes with modulated expression (log2-fold change, p<0.001) upon ORMV infection. (DOC) [file pone.0019549.s007.doc]

**Table S6. Functional annotations for genes with modulated expression (log_2_**-fold change, p<0.001) upon ORMV infection

| **ORMV vs mock** | **Annotation term** | **p-value*** |
| --- | --- | --- |
| 7dpi ∩ 14dpi ∩ 21dpi | oxylipin biosynthetic process (GO:0031408) | 3.38E-02 |
|  | oxylipin metabolic process (GO:0031407) | 3.38E-02 |
|  | defense response (GO:0006952) | 3.38E-02 |
|  | response to stimulus (GO:0050896) | 3.38E-02 |
|  | multi-organism process (GO:0051704) | 3.38E-02 |
|  | immune system process (GO:0002376) | 3.38E-02 |
|  | immune response (GO:0006955) | 3.38E-02 |
|  | jasmonic acid biosynthetic process (GO:0009695) | 3.38E-02 |
|  | jasmonic acid metabolic process (GO:0009694) | 3.38E-02 |
|  | innate immune response (GO:0045087) | 3.38E-02 |
|  | response to other organism (GO:0051707) | 3.38E-02 |
|  | response to biotic stimulus (GO:0009607) | 3.38E-02 |
|  | jasmonic acid and ethylene-dependent systemic resistance (GO:0009861) | 4.47E-02 |
|  | purine base biosynthetic process (GO:0009113) | 4.47E-02 |
|  | regulation of response to stimulus (GO:0048583) | 4.47E-02 |
| 7dpi ∩ 14dpi ! 21dpi | response to other organism (GO:0051707) | 1.01E-04 |
|  | response to stress (GO:0006950) | 1.01E-04 |
|  | response to stimulus (GO:0050896) | 1.01E-04 |
|  | defense response (GO:0006952) | 1.01E-04 |
|  | response to biotic stimulus (GO:0009607) | 2.55E-04 |
|  | multi-organism process (GO:0051704) | 6.08E-04 |
| 7dpi ∩ 21dpi ! 14dpi | response to stimulus (GO:0050896) | 4.39E-05 |
|  | immune system process (GO:0002376) | 1.44E-04 |
|  | immune response (GO:0006955) | 1.44E-04 |
|  | innate immune response (GO:0045087) | 1.44E-04 |
|  | defense response (GO:0006952) | 1.99E-04 |
|  | response to stimulus (GO:0050896) | 4.39E-05 |
| 14dpi ∩ 21dpi ! 7dpi | response to abiotic stimulus (GO:0009628) | 4.20E-10 |
|  | response to water deprivation (GO:0009414) | 2.25E-06 |
|  | response to water (GO:0009415) | 2.25E-06 |
|  | response to stimulus (GO:0050896) | 5.33E-06 |
|  | response to temperature stimulus (GO:0009266) | 7.42E-06 |
|  | response to chemical stimulus (GO:0042221) | 4.72E-05 |
|  | response to stress (GO:0006950) | 4.72E-05 |
|  | response to endogenous stimulus (GO:0009719) | 9.53E-05 |
|  | response to abscisic acid stimulus (GO:0009737) | 2.64E-04 |
|  | response to cold (GO:0009409) | 7.71E-04 |
| 7dpi ! 14dpi ! 21dpi | response to stimulus (GO:0050896) | 9.23E-14 |
|  | defense response (GO:0006952) | 1.21E-11 |
|  | response to stress (GO:0006950) | 1.46E-11 |
|  | response to chemical stimulus (GO:0042221) | 6.33E-10 |
|  | response to endogenous stimulus (GO:0009719) | 3.02E-09 |
|  | immune system process (GO:0002376) | 3.02E-09 |
|  | immune response (GO:0006955) | 3.02E-09 |
|  | response to hormone stimulus (GO:0009725) | 9.27E-07 |
|  | innate immune response (GO:0045087) | 1.08E-06 |
|  | response to ethylene stimulus (GO:0009723) | 4.93E-06 |
|  | regulation of defense response (GO:0031347) | 2.53E-05 |
|  | ethylene mediated signaling pathway (GO:0009873) | 6.35E-05 |
|  | two-component signal transduction system (phosphorelay) (GO:0000160) | 6.41E-05 |
| 14dpi ! 7dpi ! 21dpi | photosynthesis (GO:0015979) | 4.08E-07 |
|  | cellular process (GO:0009987) | 5.90E-06 |
|  | metabolic process (GO:0008152) | 5.90E-06 |
|  | cellular metabolic process (GO:0044237) | 1.31E-05 |
| 21dpi ! 7dpi ! 14dpi | response to chemical stimulus (GO:0042221) | 2.15E-09 |
|  | response to stimulus (GO:0050896) | 1.05E-08 |
|  | response to stress (GO:0006950) | 3.91E-06 |
|  | response to abiotic stimulus (GO:0009628) | 7.21E-05 |
|  | response to endogenous stimulus (GO:0009719) | 8.75E-05 |

∩, intersect; !, exclude. * Functional annotation terms have a p-value cut-off of p<0.05 for group ORMV vs. mock 7dpi ∩ 14dpi ∩ 21dpi; p<0.001 for all other groups with two intersecting time points, and p<0.0001 for groups exclusively regulated at single time points.
